# Supplementary material for: Complexome profiling of the Chlamydomonas psb28 mutant reveals TEF5 as an early PSII assembly factor
Source: Plant Cell. 2025 Mar 17;37(6):koaf055. doi: 10.1093/plcell/koaf055 (PMC12164590; doi:10.1093/plcell/koaf055)
Supplement: koaf055_Supplementary_Data [file koaf055_supplementary_data.zip › psb28_interactive.zip!/plots/intensity/qualityPlots/peptides/LHCBM1_Cre01.g066917.t1.1.html]

### Cre01.g066917.t1.1

Trivial name: LHCBM1  
SeqCoverage [median]: 41.40  
Mass: 27.57 kDa  
  
 

| Sequence | MissedCleavage | Mass [Da] | wtIntensity | muIntensity | Score |
| --- | --- | --- | --- | --- | --- |
| ASTPDSFWYGPER | 0 | 1511.67 | 78649.77 | 101327.86 | 186.24 |
| GPLQNLSDHLANPGTNNAFAYATK | 0 | 2513.23 | 1971765.16 | 2605746.98 | 260.01 |
| LYPGGSFDPLGLADDPDTFAELK | 0 | 2437.17 | 2899595.07 | 1706366.23 | 177.30 |
| LYPGGSFDPLGLADDPDTFAELKVK | 1 | 2664.33 | 106232.35 | 91583.20 | 107.56 |
| LYPGGSFDPLGLADDPDTFAELKVKEIK | 2 | 3034.55 | 936.64 | 0.00 | 33.26 |
| VNGGPLGEGLDK | 0 | 1154.59 | 2105636.50 | 1368892.13 | 226.93 |
|  |  |  | 7162815.5 | 5873916.4 |  |
